# Epidemiology of *Mycobacterium tuberculosis* complex infections in cattle and humans in the remote pastoral settings of southern Ethiopia

**DOI:** 10.3389/fvets.2025.1551710

**Published:** 2025-03-19

**Authors:** Temesgen Mohammed, Fekadu Desta, Biniam Wondale, Aboma Zewude, Gezahegne Mamo, Hazim O. Khalifa, Berecha Bayissa, Gobena Ameni

**Affiliations:** ^1^Department of Veterinary Medicine, College of Agriculture and Veterinary Medicine, United Arab Emirates University, Al Ain, United Arab Emirates; ^2^Aklilu Lemma Institute of Pathobiology, Addis Ababa University, Addis Ababa, Ethiopia; ^3^Department of Biology, Arba Minch University, Arba Minch, Ethiopia; ^4^College of Veterinary Medicine and Agriculture, Addis Ababa University, Debre Zeit, Ethiopia; ^5^Faculty of Veterinary Medicine, Kafrelsheikh University, Kafr El-Sheikh, Egypt; ^6^Vaccine Production and Drug Formulation Directorate, National Veterinary Institute, Bishoftu, Ethiopia

**Keywords:** *Mycobacterium tuberculosis* complex, mycobacterial lineage, pastoralists, prevalence, risk factor, southern Ethiopia

## Abstract

**Introduction:**

*Mycobacterium tuberculosis* complex (MTBC) infections are characterized by the development of granulomatous lesions in different parts of the bodies of animals and humans. MTBC infections cause significant economic and public health consequences in Ethiopia. However, there is a shortage of epidemiological data on MTBC infections in the pastoral regions of the country. The objective of the present study was to investigate the epidemiology of MTBC infections in cattle and humans in the remote pastoral setting of southern Ethiopia.

**Methods:**

A cross-sectional study design was used to recruit 2,396 cattle and 1,200 human presumptive tuberculosis (TB) cases for this study from the southern pastoral districts of Ethiopia. The single intradermal comparative cervical tuberculin test (SICCTT) was used to screen for bovine TB in the cattle, while mycobacterial culture and spoligotyping were used to identify mycobacterial species and strains in the pastoralists.

**Results:**

The herd and animal prevalences of bovine TB were 14.9% [95% confidence interval (CI) = 10.2–19.5%] and 3.2% (95% CI: 2.5–4.0), respectively. The herd prevalence was associated with the districts (*χ*^2^ = 40.10, *p* < 0.001). Based on the multivariable binary logistic regression analysis, the male animals were 1.77 (95% CI: 1.02–3.05) times more likely to be TB positive than the female animals. Similarly, the cattle kept in the Dasenech and Benetsemi districts were 10.65 (95% CI: 2.47–45.87) and 22.94 (95% CI: 5.48–95.94) times more likely to be TB positive than the cattle kept in the Selamago district, respectively. Mycobacterial culture positivity was 13.4%, while spoligotyping identified Euro-American (EA), East African-Indian (EAI), Indo-Oceanic (IO), lineage 7, *M. bovis,* and *M. africanum* as the major lineages, with proportions of 67.3% (105/156), 22.4% (35/156), 6.4% (10/156), 1.9% (3/156), 1.3% (2/156), and 0.6% (1/156), respectively.

**Conclusion:**

In general, the prevalence of bovine TB was relatively lower than that recorded in intensive dairy farms in central Ethiopia. Three species of MTBC, namely *M. tuberculosis*, *M. africanum,* and *M. bovis,* were isolated from the pastoralists of southern Ethiopia. The isolation of *M. bovis* from the pastoralists could suggest its zoonotic transmission from cattle to humans.

## 1 Introduction

*Mycobacterium tuberculosis* complex (MTBC) causes mycobacterial diseases in animals and humans. Mycobacterial infections are characterized by the development of progressive granulomatous lesions in different tissues. The major disease-causing members of MTBC are *M. tuberculosis*, *M. bovis*, *M. caprae,* and *M. africanum* (1). Bovine tuberculosis (TB) is primarily caused by *M. bovis* and is a World Organization for Animal Health (WOAH)-listed disease. Bovine TB affects cattle health, reduces the productivity of infected cattle, restricts the trading of cattle, and affects genetic improvement (2). In addition, although *M. tuberculosis* is the major cause of human TB, *M. bovis* is transmitted to humans through the consumption of contaminated animal products or the inhalation of aerosol droplets, causing a significant public health problem (3). Historical data have indicated a direct correlation between *M. bovis* infection in cattle and human populations (4). However, there is a shortage of data on the epidemiology of mycobacterial infections in cattle and humans in developing countries (5).

According to the WOAH (6), bovine TB remains endemic and largely uncontrolled in Africa, Asia, Latin America, and most countries in the Middle East. Bovine TB is a legally notifiable disease in livestock in several African countries (7), although it is widespread in livestock at the continental level (8). In the East African region, existing evidence generally suggests a low prevalence of bovine TB in both wild and domestic animals (9), although high prevalence values have been recorded in intensive husbandry systems in Ethiopia, Uganda, and Tanzania (10). In addition, high prevalence was reported in pastoral livestock in Uganda (11).

In Ethiopia, individuals living close to the livestock–human interface and consuming mostly unpasteurized milk and dairy products are at risk of infection with *M. bovis* (8). In addition, infection with HIV/AIDS facilitates the transmission and progression of TB in humans. Subsequently, studies have shown a significantly increased proportion of *M. bovis* infections in individuals co-infected with HIV and TB (12–14). This observation highlights the role of *M. bovis* in public health in countries endemic to bovine TB (15), although Ethiopian authors have reported a low prevalence of *M. bovis* in human extrapulmonary TB cases (16).

Epidemiological data have identified poor or absent control strategies, uncontrolled movements of animals, lack of herd screening for bovine TB, increased contact between different herds, and contact with wild species as risk factors for the transmission of bovine TB (17, 18). Pastoralists and their animals are considered to be at high risk of contracting TB because of their lifestyles. Animals congregate on grazing lands and watering points, which creates a conducive environment for the transmission of TB among animals. In addition, pastoralists live in close contact with their animals and also consume raw milk, which facilitates the transmission of TB from animals to pastoralists (19). A few studies have been conducted on TB in the pastoral zones of Ethiopia (20–22). *M. bovis*, *M. tuberculosis,* and NTM were isolated as causative agents of TB in animals in the pastoral zones of Ethiopia (23, 24). Additional studies are required on mycobacterial infections in animals and pastoralists in a comprehensive way so that adequate data are generated and used for planning appropriate control strategies. Therefore, the objective of the present study was to investigate the epidemiology of MTBC infections in cattle and pastoralists in the remote pastoral setting of southern Ethiopia.

## 2 Materials and methods

### 2.1 Study area

**Figure 1:**
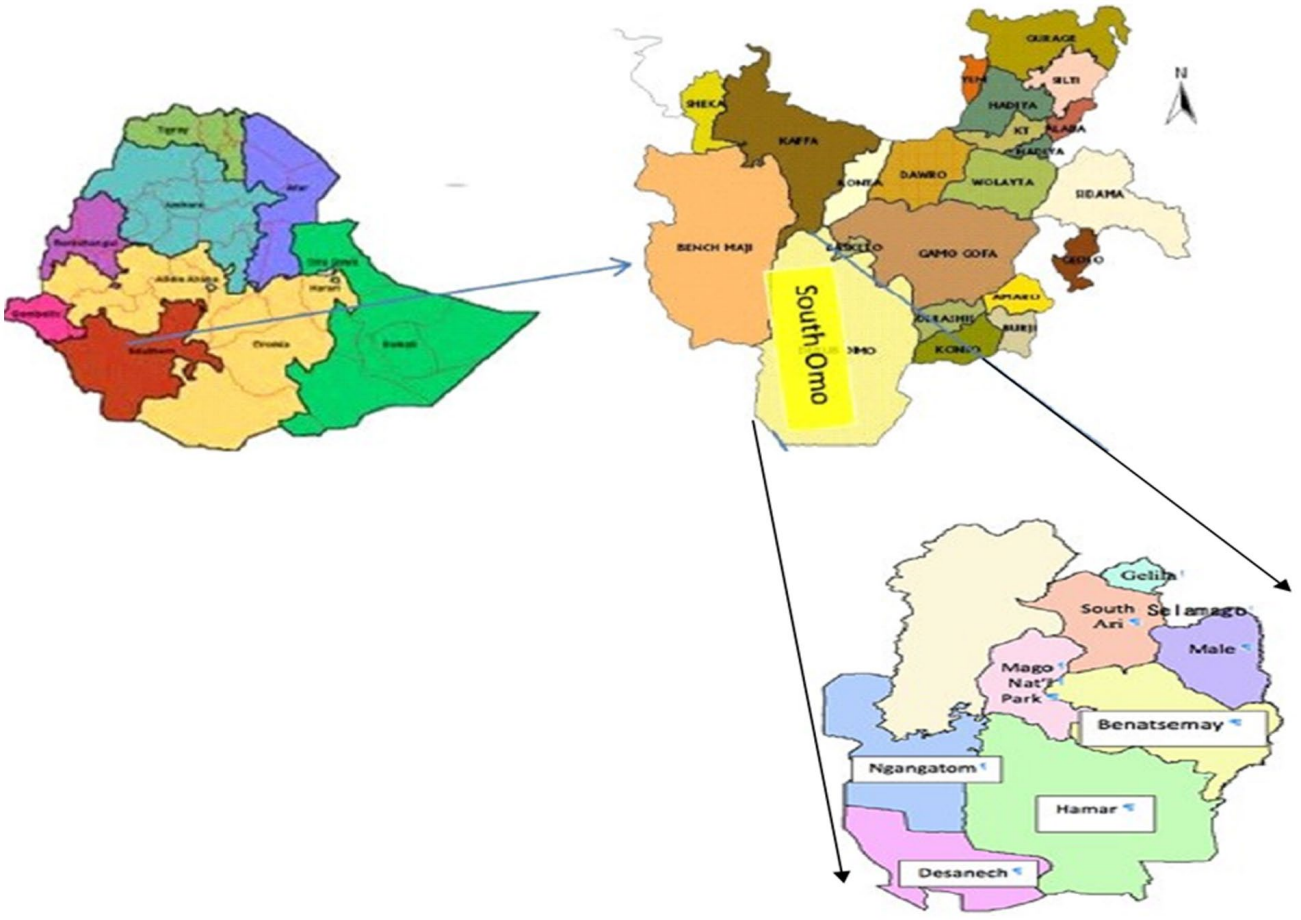
Study districts in South Omo Zone. South Omo Zone is located in the Southern Nations, Nationalities, and People’s Region, Ethiopia. Reproduced with permission from Enyew (27).

This study was conducted in the South Omo Zone of southern Ethiopia, located near the border with Kenya (25), as shown in Figure 1. The administrative center of the Zone is located in Jinka town. The zone was named after the Omo River, which flows through the zone into Lake Turkana in Kenya. South Omo Zone is the most sparsely populated part of Ethiopia, inhabited by 16 ethnic groups (25). The livestock found in the South Omo Zone include cattle, sheep, goats, donkeys, camels. Accordingly, the livestock population of the study area is estimated as follows: cattle 1,134,120; sheep 489,449; goats 1,230,399; donkeys 90,630; camels 495; poultry 424,538; and bee colonies 132,500 (South Omo Zone Agricultural and Rural Development Offices, 2014).

### 2.2 Study design, sampling method, and sample size determination

A cross-sectional study was carried out from August 2020 to December 2022 on the indigenous zebu cattle found in the remote pastoral setting of southern Ethiopia. Five districts were included in the study, and settlements (villages) in each sub-district were selected based on the inclusion criteria (accessibility, security, and willingness of pastoralists to participate in the research) and after obtaining consent from the responsible body. Cattle herds were selected using cluster sampling, where each cattle herd unit was considered a cluster. The sample size was calculated using the cluster sampling formula, as described by Bennett et al. (26). The initial sample size was determined based on a 95% confidence level, a 0.05 margin of error, and a 0.8% prevalence, as described by Tschopp et al. (9), resulting in a sample size of 246 per district. The design effect was calculated using the formula from Bennett et al. (26), with a rho(p) value of 10%, and 15 animals were selected from each cluster. Consequently, the total sample size was calculated to be 600 animals per district from 40 clusters, which resulted in a total sample size of 3,000 animals across five districts. However, as not all owners availed their animals during the second reading, complete results could not be obtained for all the 600 animals per district.

**Figure 2:**
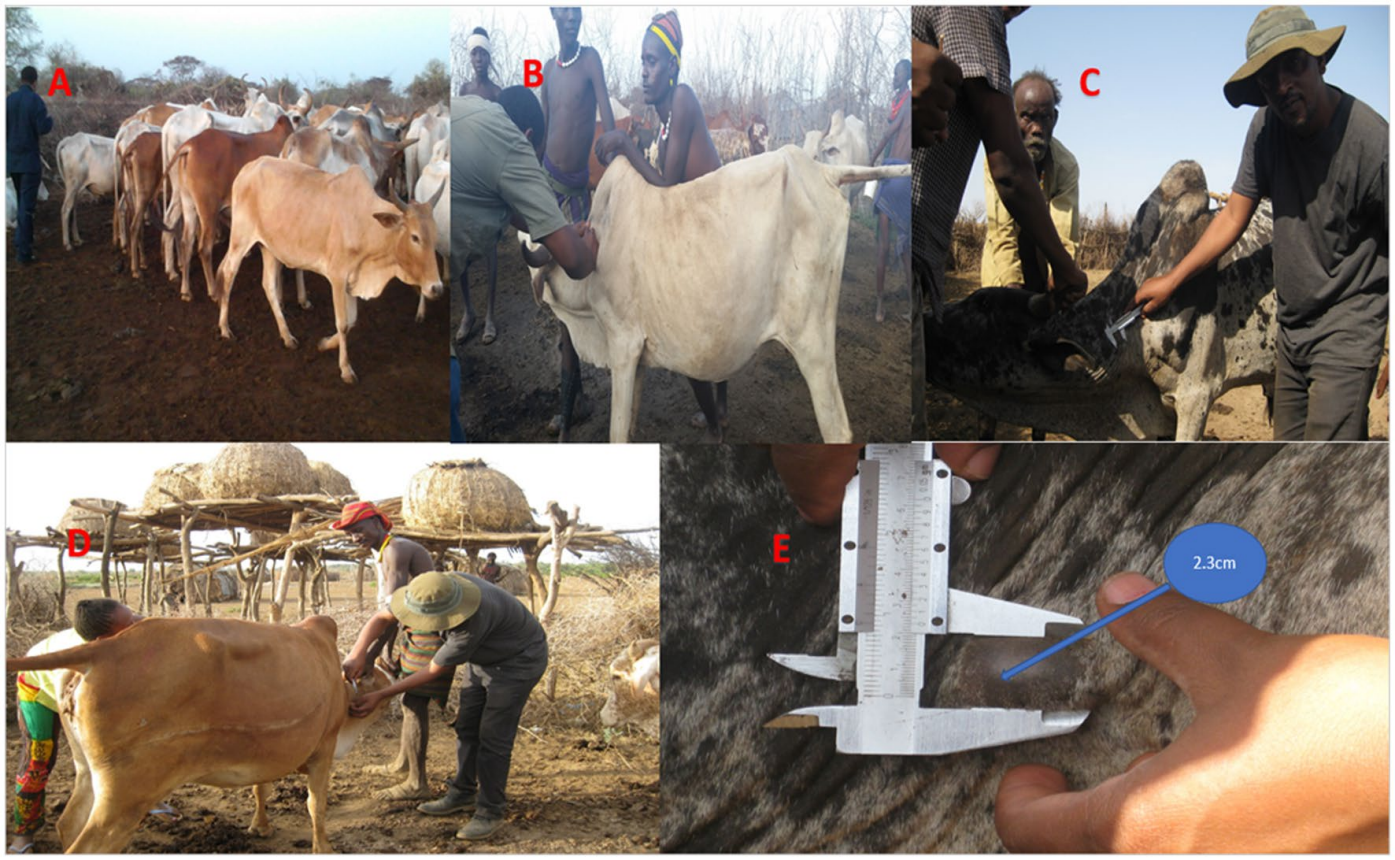
Single intradermal comparative cervical tuberculin test. **(A)** Gathering of the tested cattle in one place**. (B)** Shaving of two sites on the middle-left side of the neck. **(C)** Measuring the skin thickness with a caliper. **(D)** Injection of aliquots of 0.1 mL of avian purified protein derivative (PPD) and 0.1 mL of bovine PPD into the dermis of the cattle. **(E)** Measuring the skin thickness at each injection site after 72 h.

Regarding the sampling, a complete list of sub-districts and villages within the sub-districts was obtained from each district agricultural office. Since the cattle of all villagers were kept together during the day, at least during grazing and watering, each village was considered one large herd for the assessment of tuberculin reactivity status. Herd selection was made proportionally to represent each cluster within the large herd. Thus, cattle owned by one or more owners, where the animals shared common grazing sites and watering points, were kept at night at a common site (Figure 2), and moved together during migration, were considered a herd. Animals younger than 6 months of age were excluded. The demographic data of each study animal were recorded. All animals included in the study were dewormed. In total, 2,396 cattle that fulfilled the inclusion criteria were tested using the SICCTT.

**Figure 3:**
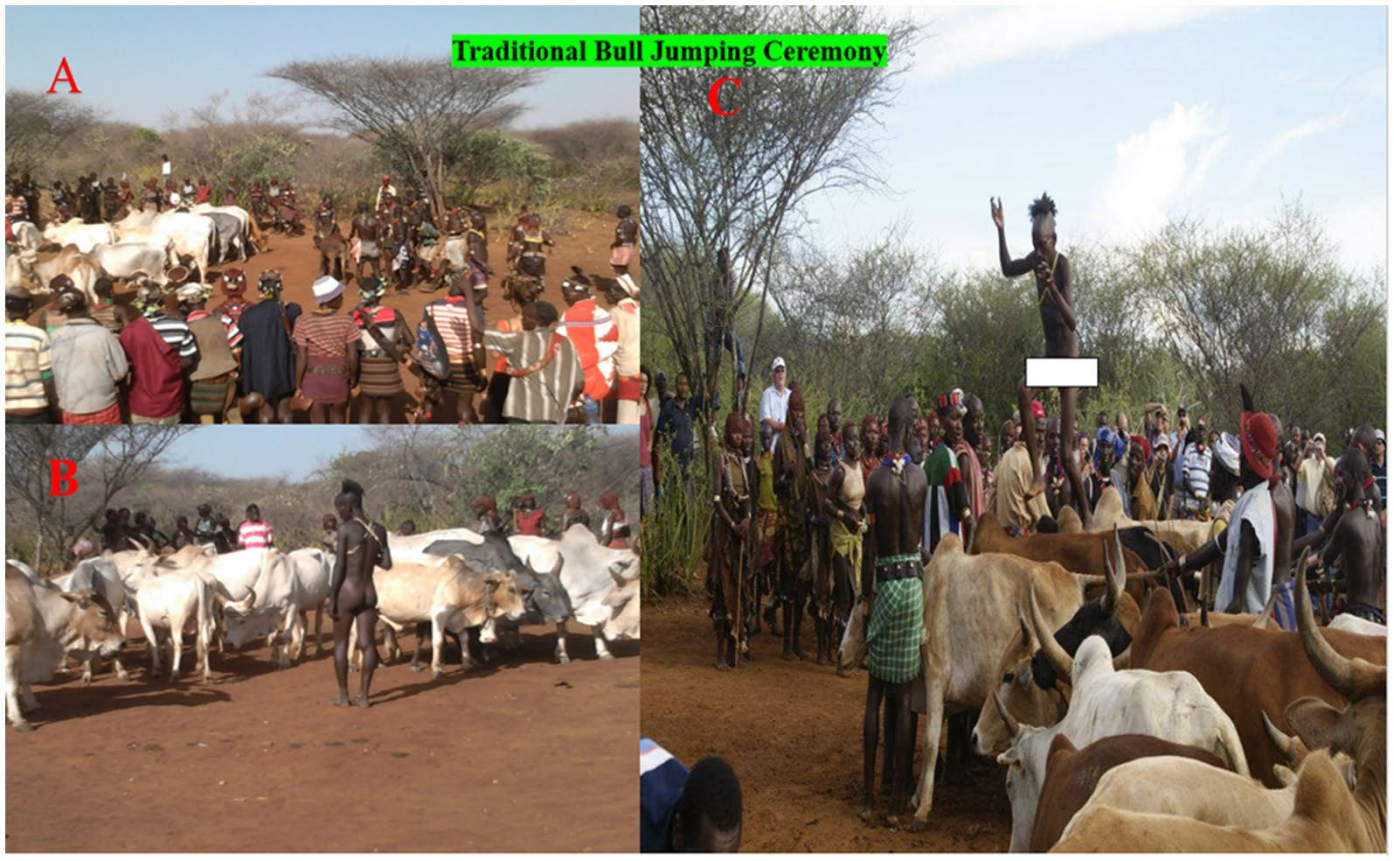
Hammer bull jumping ceremony. **(A)** Gathering of the male cattle at the ceremony location**. (B)** Lining up of the cattle in a row by the men of the tribes **(C)** Leaping up onto and running over the deep row of lined cattle.

On the other hand, sample size calculation was not performed to estimate the sample size for human participants, as the objective of the human study was to evaluate the transmission of MTBC between cattle and humans. Since cattle are central to the various social events of the South Omo Zone pastoralists, such as those of the Hammer pastoralists, the contact between cattle and humans is very frequent (Figure 3). This contact is expected to facilitate the transmission of mycobacterial infections between humans and cattle. Due to security and logistic problems, it was not possible to test the owners of the cattle with bovine TB. Instead, all human presumptive TB cases were recruited and used for bacterial isolation and identification to estimate the burden of *M. bovis* as a causative agent of human TB in the South Omo Zone. Accordingly, 1,200 presumptive cases were recruited and used for sample collection.

### 2.3 Data collection

Demographic data for each study cattle (sex, age, body condition score, BCS) were collected when the study animals were tested for bovine TB. The ages of the cattle were determined using dental eruption and wear according to the established procedure (28). The body condition of each of the study cattle was scored using previously established guidelines (29).

### 2.4 Single intradermal comparative cervical tuberculin test

The single intradermal comparative cervical tuberculin test (SICCTT) was performed using both bovine purified protein derivative (PPD) and avian PPD obtained from Prionics, Lelystad, The Netherlands (Figure 2). Two sites on the middle-left side of the neck, separated by 12 cm, were shaved, and skin thicknesses were measured with a caliper. Aliquots of 0.1 mL of 2,500 IU per ml of the avian PPD and 0.1 mL of 2000 IU per ml of the bovine PPD were injected into the dermis of the study animals. Subsequently, the skin thickness at each injection site was measured again after 72 h. The interpretation of the result was based on the recommendation of the WOAH (30).

### 2.5 Collection of sputum and fine needle aspirates for mycobacterial culturing

Health centers and a hospital providing healthcare services in the study zone were used for the recruitment of presumptive TB cases and the collection of samples for mycobacterial culture. Sputum samples were collected on a regular basis when patients submitted samples for the routine diagnosis of TB. In addition, fine needle aspirates (FNAs) were collected from the extrapulmonary TB cases by a pathologist. In total, 1,200 samples (1,166 sputum and 34 FNA samples) were collected for laboratory analysis. Both the sputum and FNA samples were stained with the Ziehl–Neelsen stain for the detection of acid-fast bacilli. The remaining samples were transported to the TB laboratory at Jinka hospital, in a cold chain for culturing. The samples were stored at 20°C in a freezer until thawed and processed for culturing.

The FNA samples were directly inoculated onto the LJ medium, while the sputum samples were processed (decontaminated and neutralized) for culturing according to the standard operating procedure described earlier (31). In brief, an equal volume of 4% NaOH was mixed with the sputum samples, and the mixture was centrifuged at 3000 rpm for 15 min at room temperature. After decanting the supernatant, the sediment was neutralized with 2 N HCl using phenol red as an indicator. Neutralization was achieved when the color of the solution changed from purple to yellow. Thereafter, 100 μL of the suspension was inoculated onto two sterile LJ medium slopes, which were enriched with either pyruvate or glycerol. The inoculated media were then incubated at 37°C in a slanted position for 1 week and in an upright position for 4–5 weeks. The growth of mycobacteria was monitored every week for up to 8 weeks. Slants with no evidence of growth after 8 weeks were considered negative (32). Specimens with colony growth were examined for acid-fast bacilli (AFB) after staining with the Ziehl–Neelsen stain.

### 2.6 DNA extraction and spoligotyping

The colonies positive for acid-fast bacilli (AFB) were harvested and re-suspended in 200 μL sterile distilled water. Afterward, the suspension was inactivated by heating at 80°C for 45 min in a water bath to release the DNA. Spoligotyping was performed following the procedure described by Kamerbeek et al. (33). The laboratory results of spoligotyping were interpreted in binary format, and lineages were assigned using an updated version of SITVITWEB (34). Major lineages were assigned using the “TB insight” database. Isolates that exhibited similar patterns to those in the SITVIT database were assigned shared international types (SITs), i.e., SIT numbers. Isolates not assigned to shared international types (i.e., SIT numbers) were referred to as “Orphans.”

### 2.7 Ethical consideration

The study obtained ethical clearance from the Aklilu Lemma Institute of Pathobiology (ALIPB), Addis Ababa University (Ethiopia), the Institutional Review Board (ALIPB/IRB/22-B/2012/13), and the National Research Ethics Committee (Ref no. 3.10/785/07). Permission to conduct the study was also obtained from the South Omo Zone Health Department. Written informed consent was obtained from each of the study participants, and for those under 18 years of age, consent was also obtained from their parents or guardians.

### 2.8 Data management and analysis

The data were coded using a Microsoft Excel sheet and transferred and analyzed using SPSS version 28 (IBM SPSS Statistics). Pearson’s chi-squared test was used to evaluate the statistical significance of the associations between different categorical variables and the prevalence of bovine TB. Univariable and multivariable binary logistic regression analyses were used to estimate the strength of the associations between the risk factors and the prevalence of bovine TB. A *p* < 5% was considered statistically significant. When estimating the effect of the different risk factors in terms of odds ratio (OR) with the corresponding 95% confidence interval, statistical significance was assumed when the confidence interval did not include one.

## 3 Results

### 3.1 Herd-level prevalence of bovine tuberculosis

The herd prevalence of bovine TB was 14.9% (95% CI: 10.2–19.5%) in the South Omo Zone. The herd prevalence significantly differed among the five districts of South Omo Zone (*χ*^2^ = 40.9; *p* < 0.001), while there was no significant difference in the herd prevalence across different herd sizes (*p* > 0.05) (Table 1).

**Table 1:** Herd prevalence of bovine TB and associated risk factors in South Omo zone of southern Ethiopia.

Risk factors	Category	Status of bTB		Total	Chi-square	*p*-value
		Negative	Positive			
Herd size	≤5	61	11	72	2.39	0.303
	6–60	59	14	73		
	≥61	63	7	70		
District	Hammer	5	4	9	40.09	< 0.001
	Dasenech	0	3	3		
	Mali	87	1	88		
	Benetsemi	86	22	108		
	Selamago	5	2	7		

### 3.2 Animal-level prevalence of bovine TB and associated host risk factors

The animal prevalence of bovine TB was 3.2% (95% CI: 2.5–4.0) in the South Omo Zone. The animal prevalence differed by age (*p* < = 0.05), sex (*p* < 0.01), body condition score (*p* < 0.05), and districts (*p* < 0.001) (Table 2).

**Table 2:** Animal prevalence of bovine TB and associated risk factors in South Omo zone of southern Ethiopia.

Risk factors	Category	Status of bTB		Total	Chi-square	*p*-value
		Negative	Positive			
Age	<2 years	92	1	93	10.69 (Fisher’s Exact test)	0.011
	2–5 years	819	15	834		
	5–9 years	1,149	48	1,197		
	> 9 years	260	12	272		
Sex	Male	701	34	735	7.3	0.008
	Female	1,619	42	1,661		
Body condition	Poor	347	6	353	7.1	0.028
	Moderate	1,625	64	1,689		
	Good	348	6	354		
District	Hammer	404	4	408	85.49	< 0.001
	Dasenech	419	39	458		
	Mali	500	1	501		
	Benetsemi	516	30	546		
	Selamago	481	2	483		

The results of the univariable binary logistic regression analysis of the association between the risk factors and the animal prevalence are presented in Table 3. The univariable binary logistic regression analysis showed that the cattle aged between 5 and 9 years and those aged above 9 years were 4.25 (95% CI: 0.55–33.11) and 1.87 (95% CI: 1.18–2.96) times more likely to test positive for bovine TB than the cattle younger than 2 years, respectively.

**Table 3:** Association of animal prevalence of bovine TB with risk factors based on univariable binary logistic regression analysis.

Risk factors	Category	Status of bTB		Total	COR (95% CI)	*p*-value
		Negative	Positive			
Age	<2 years	92	1	93	1	0.020
	2–5 years	819	15	834	1.69 (0.22–12.90)	
	5–9 years	1,149	48	1,197	3.84 (0.53–28.16)	
	> 9 years	260	12	272	4.25 (0.55–33.11)	
Sex	Male	701	34	735	1.87 (1.18–2.96)	0.008
	Female	1,619	42	1,661	1	
BCS	Poor	347	6	353	1.0 (0.32–3.14)	0.035
	Moderate	1,625	64	1,689	2.28 (0.98–5.32)	
	Good	348	6	354	1	
District	Hammer	404	4	408	2.38 (0.43–13.07)	< 0.001
	Dasenech	419	39	458	22.39 (5.37–93.26)	
	Mali	500	1	501	0.48 (0.04–5.32)	
	Benetsemi	516	30	546	13.98 (3.32–58,82)	
	Selamago	481	2	483	1	

The results of the multivariable binary logistic regression analysis are presented in Table 4. According to the multivariable binary logistic regression analysis, the male animals were 1.77 (95% CI: 1.02–3.05) times more likely to be TB positive compared to the female animals. Similarly, the cattle kept in Dasenech and Benetsemi were 10.65 (95% CI: 2.47–45.87) and 22.94 (95% CI: 5.48–95.94) times more likely to be TB positive than the cattle kept in Selamago, respectively.

**Table 4:** Multivariate logistic regression analysis of tuberculin reactors with various host-related risk factors at 4 mm cut-off point.

Risk factor	Category	Total cattle examined	Total cattle Positive (%)	AOR (95% CI)	*p*-value
Age (years)				–	0.039
	<2 years	93	1 (1.1)	1	–
	2–5 years	834	15 (1.8)	1.28 (0.15–9.27)	0.878
	5–9 years	1,197	48 (4)	2.63 (0.35–19.7)	0.347
	> 9 years	272	12 (4.4)	3.11 (0.38–24.90)	0.287
Sex	Male	735	34 (4.60)	1.77 (1.02–3.05)	0.041
	Female	1,661	42 (2.5)	1	–
District					< 0.0001
	Hammer	408	4 (0.98)	2.65 (0.48–14.67)	0.265
	Dasenech	458	39 (8.5)	22.94 (5.48–95.94)	< 0.0001
	Mali	501	1 (0.2)	0.50 (0.05–5.56)	0.570
	Benetsemi	546	30 (5.5)	10.65 (2.47–45.87)	0.001
	Selamago	483	2 (0.4)	1	–

BCS, Body condition scoring.

### 3.3 Isolation and identification of mycobacteria from human participants

A total of 1,200 human presumptive TB cases (1,166 pulmonary TB and 34 extrapulmonary TB cases) were identified by healthcare providers and then included in this study. Smear positivity was observed in 21.3% (255/1200) of the presumptive cases. Acid-fast bacilli were confirmed in all of the 34 extrapulmonary TB cases and 221 pulmonary TB cases. The overall prevalence of culture positivity was 13.4% (161/1200). Culture positivity was 76.5% (26/34) in the FNA samples, while it was 11.6% in the pulmonary TB cases (135/1166). The isolation rate of *M. bovis* was 1.2% (two out of 161 isolates). The two *M. bovis* isolates were from the pulmonary TB cases.

Spoligotyping was performed on 156 of the 161 culture-positive DNA samples (Table 5), identifying 66 different patterns (24 clustered and 42 single strains). The majority of the isolates (76.3%) had shared international types (SITs), while the remaining 23.7% were orphan strains. The most common major lineages identified were Euro-American (EA), East African-Indian (EAI), and Indo-Oceanic (IO), with proportions of 67.3% (105/156), 22.4% (35/156), and 6.4% (10/156), respectively. Lineage 7, *M. bovis,* and *M. africanum* were isolated, with proportions of 1.9% (3/156), 1.3% (2/156), and 0.6% (1/156), respectively.

**Table 5:** Spoligotype patterns of *M. bacterium tuberculosis* complex species isolated from human in the South Omo zone of southern Ethiopia.

CBN* Lineage	Isolates no.	Shared types (SIT numbers)	Orphan strains
Euro-American	105	81	24
East-African-Indian	35	29	6
Indo-oceanic	10	5	5
Linage_7	3	3	0
*M. bovis*	2	1	1
*M. africanum*	1	0	1
Total	156	119	37

## 4 Discussion

The present study was conducted in the remote South Omo Zone pastoral districts of southern Ethiopia on mycobacterial infections in cattle and pastoralists. Herd and animal prevalences of bovine TB were estimated, and lineages of MTBC members were identified from pastoralists. Two *M. bovis* were isolated from the sputum samples of the human TB cases.

Bovine TB is endemic in Ethiopia (35), and over the last two decades, a significant number of studies have been conducted, primarily in the central zones of the country, while only a few studies have been conducted in the pastoral zones (36). In this context, the present study is considered an addition to the limited studies conducted on humans and animals in the pastoral zones. The animal prevalence recorded in this study was low in general; however, it was higher than the animal prevalence values recorded in other pastoral zones of Ethiopia (9, 24, 37). Furthermore, it was lower than the values reported by other researchers in different pastoral areas of Ethiopia (21, 38–40). The difference in the prevalence of bovine TB across different pastoral zones could be attributed to factors such as herd size, the availability of adequate grazing pasture, the extent of co-grazing among different herds, and the magnitude of interaction with wildlife. These factors also affect the herd prevalence of bovine TB. The observed herd prevalence was similar to the herd prevalence reported earlier by other researchers in different pastoral zones of Ethiopia (24, 41). However, it was lower than the herd prevalence values reported in other studies (21, 38, 42–45). In contrast, some other studies reported herd prevalence values lower than those recorded in this study (46, 47).

The animal prevalence was associated with age, sex, body condition, and the districts in which the cattle were kept. Similar observations were previously reported in Ethiopia (21, 38, 39, 48, 49), Ecuador (50), and India (51). Consistent with the findings of this study, other studies also reported an association between animal prevalence and the body condition of cattle (44, 49, 52). The animal prevalence of bovine TB increased with the age of the cattle, as indicated by the multinomial binomial logistic regression analysis. This observation was also previously reported by other researchers (15, 53–58). The probable reason for this is that older animals have had repeated opportunities for exposure to mycobacterial infections throughout their lifetimes. In addition, age-related immune suppression makes older animals more susceptible to mycobacterial infections (59). The results of this study showed that the animals with moderate body condition were at a higher risk of having BTB than the animals with poor body condition. It is known that the SICCTT is influenced by the immune status of animals. Animals with poor body condition are prone to false-negative results because of their weak immune responses to the tuberculin injection. The weak immune response to the tuberculin injection could be due to malnutrition, the advanced stage of TB causing a shift in the immune response from Th1 to Th2, or parasite infestation, which can also lead to a shift in the immune response from Th1 to Th2. In addition, the results of this study showed that the male animals were at a higher risk of being reactors to the SICCTT compared to the female animals. This difference in reactivity between male and female animals could be due to their physiological differences, which could affect their immune responses to the tuberculin injection. Female animals could be either pregnant or lactating, both of which can compromise their responses to the tuberculin injection.

The low prevalence of bovine TB could also be due to the low sensitivity of the SICCTT. Furthermore, exposure to environmental mycobacteria, infection with *Mycobacterium avium* subspecies *paratuberculosis* (MAP), infestation with intestinal parasites, and malnutrition could further lower the sensitivity of the SICCTT, potentially resulting in a high rate of false-negative animals. The high rate of false-negative cases could also contribute to the low prevalence. Therefore, the SICCTT should be supplemented by other tests, such as the interferon gamma test and serological tests, to increase the detection rate. Particularly, parallel testing of study animals with the SICCTT and a rapid serological test allows for the detection of animals both in the early and advanced stages of bovine TB, thereby increasing detection sensitivity.

Differences were observed in the prevalence of bovine TB among the five study districts. The prevalence was relatively higher in the Dasenech and Benetsemi districts. This difference could be due to factors such as cattle population density, the degree of interaction between cattle from different herds and wild animals, the prior introduction of infected cattle to the district, and the nutritional and health status of cattle, as well as environmental and climatic factors (53).

The proportion of culture positivity in the sputum samples was very low. Other researchers have reported relatively higher proportions of culture positivity in Ethiopia (60, 61), although one study reported a similarly low proportion of sputum culture positivity (62). The low proportion of sputum culture positivity was mainly associated with issues in sample handling. Due to the harsh environment and remote location, the samples could not be maintained at the optimal temperature during collection. The cold chain was used, but it was not easy to replace the ice packs. In addition, the sputum samples were stored for approximately 1 month at the field sites before being transported to the laboratory for culturing. These sample handling conditions could kill the bacilli, and since culture positivity is directly related to the availability of bacilli in the sputum, such poor sample handling procedures could lead to low sputum culture positivity.

Based on spoligotyping, six lineages were isolated from the pastoralists—Euro-American, EAI, Indo-Oceanic, lineage 7, *M. bovis,* and *M. africanum*. The dominant lineage, the Euro-American lineage, constituted 67.3% of the total isolates. Similar observations have been reported in earlier studies (63–66). This highlights the rapid spread of the Euro-American lineage from central Ethiopia to the peripheral areas of the country. The second, third, fourth, fifth, and sixth lineages, isolated in decreasing order of frequency, included EAI, Indo-Oceanic lineage, lineage 7, *M. bovis,* and *M. africanum,* respectively. Other studies have also isolated these lineages with varying frequencies across different regions of Ethiopia (63–65, 67). *M. africanum* has been reported as a significant cause of human TB in West African countries (68–70).

Two *M. bovis* isolates were obtained from the pastoralists. Although the proportion of the *M. bovis* isolates was low, it highlights the significance of zoonotic TB in the South Omo Zone. Animals play a significant role in the livelihoods of pastoralists. In addition, animals are central to various social events for pastoralists. For example, in the South Omo Zone, a young Hammer male is expected to participate in bull jumping before marriage (Figure 3). The bull jumping ceremony is performed by every young Hammer boy before marriage. During this event, people from different villages gather to celebrate the ceremony. Cattle are also gathered together in the same place as the people and are used for jumping, as demonstrated in Figure 3. This gathering of cattle and humans during such and other social events is expected to facilitate the transmission of MTBC species between humans and cattle. In addition to physical contact between cattle and pastoralists, pastoralists consume raw milk and undercooked meat, which promote the transmission of *M. bovis* from animals to humans.

In agreement with the present study, other studies have also reported the isolation of *M. bovis* from humans in Ethiopia (16, 24, 46). These studies have reported a low percentage of *M. bovis* isolates among humans in Ethiopia. Therefore, the results of this study and previous studies suggest the minimal role of *M. bovis* in causing human TB in Ethiopia. This minimal role could be due to the fact that over 95% of the cattle in Ethiopia are of the Zebu breed, which is kept extensively on pasture. The prevalence of bovine TB is low in the Zebu breed and in cattle grazing on pasture. Therefore, since the prevalence of bovine TB is low in the Zebu breed, the rate of its transmission to humans is also low (46).

Based on spoligotyping, the clustering rate was 57.7%%, which is similar to the report of a previous study conducted in Gambella, southwest Ethiopia (71). However, the clustering rate reported in this study was lower than the rate reported by the national survey (72) and studies from Addis Ababa (73), northwest Ethiopia (74), eastern Ethiopia (75), and central Ethiopia (67). On the other hand, the clustering rate reported in this study was slightly higher than that reported from western Ethiopia (76). The difference in the clustering rate among different studies could be due to variations in population density and the interaction of people through socio-economic activities (77). In general, the clustering rate recorded in this study can be considered low, which could be due to the low density of the population in the South Omo Zone. The study districts are sparsely populated, and the interaction among the population is limited.

Nonetheless, it is important to highlight the poor discriminatory power of spoligotyping in genotyping MTBC. This limitation of spoligotyping has been discussed in previous publications (78, 79). In addition, several authors have evaluated the discriminatory power of spoligotyping, and based on the results of their studies, concluded that spoligotyping demonstrates low discriminatory power in genotyping MTBC species compared to the other genotyping tools (78, 80, 81). For this reason, Comas et al. (78) recommended combining spoligotyping with mycobacterial interspersed repetitive unit (MIRU)-based variable number tandem repeats (VNTRs) typing for the initial exploratory screening of strains.

## 5 Limitations of the study

One of the objectives of the study was to investigate the zoonotic transmission of tuberculosis between cattle and pastoralists. However, the isolation and identification of mycobacteria could not be done directly from the pastoralists and their cattle. The cattle and human studies were performed separately in the same geographic districts. The cattle study involved testing animals in the field, while the human study was conducted at a hospital and health centers on presumptive human TB cases who visited these healthcare institutions seeking treatment. Therefore, the study could not show direct human–animal transmission. On the other hand, since these human cases were residents of the area where the study cattle were raised, it was assumed that the isolation of *M. bovis* from these human cases might suggest the transmission of *M. bovis* from cattle to humans.

## 6 Conclusion

The present study revealed a low prevalence of bovine TB in the remote pastoral setting of southern Ethiopia. In addition, the isolation rate of *M. bovis* from human TB cases in the same setting was low. The low prevalence of bovine TB could be due to the low sensitivity of the SICCTT, exposure of the study animals to environmental mycobacteria, infection with MAP, infestation with intestinal parasites, and malnutrition which could affect the sensitivity of SICCTT, leading to a high rate of false-negative animals. Therefore, to improve the detection of bovine TB, it is recommended to use the SICCTT in parallel with a rapid serological test. The role of *M. bovis* in human TB cases should be investigated, particularly among pastoralists who have direct contact with infected animals and those who consume raw milk from infected animals.

## Data Availability

The raw data supporting the conclusions of this article will be made available by the authors, without undue reservation.
